# Successful antibiotic treatment of pulmonary disease caused by *Mycobacterium abscessus* subsp. *abscessus* with C-to-T mutation at position 19 in *erm*(41) gene: case report

**DOI:** 10.1186/s12879-016-1554-7

**Published:** 2016-05-17

**Authors:** Su-Young Kim, Sung Jae Shin, Byeong-Ho Jeong, Won-Jung Koh

**Affiliations:** Division of Pulmonary and Critical Care Medicine, Department of Medicine, Samsung Medical Center, Sungkyunkwan University School of Medicine, Irwon-ro 81, Gangnam-gu, Seoul, 06351 South Korea; Department of Microbiology, Yonsei University College of Medicine, Seoul, South Korea

**Keywords:** Nontuberculous mycobacteria, *Mycobacterium abscessus*, Lung diseases, Clarithromycin, Drug resistance

## Abstract

**Background:**

*Mycobacterium abscessus* complex (MABC) is the most drug resistant of the mycobacterial pathogens. *M. abscessus* subsp. *abscessus* encodes a functional erythromycin ribosomal methylase gene, *erm*(41), causing inducible macrolide resistance. However, some clinical isolates of *M. abscessus* subsp. *abscessus* harboring nonfunctional *erm*(41) were susceptible to macrolide, even after extended incubation of 14 days. Loss of function of the *erm*(41) genes was associated with a T-to-C substitution at position 28 of the gene (T28C), leading to an amino acid change from Trp to Arg at codon 10. Pulmonary disease caused by *M. abscessus* subsp. *abscessus* strains with an nonfunctional *erm*(41) (C28 sequevar) may be responsive to macrolide-containing antibiotic regimens. Therefore, all *M. abscessus* subsp. *abscessus* strains with a functional *erm*(41) (T28 sequevar) were thought to be resistant to macrolide with extended incubation. Here, we report the first case of pulmonary disease caused by a strain of *M. abscessus* subsp. *abscessus* which was susceptible to macrolide due to T19 sequevar of *erm*(41) gene.

**Case presentation:**

A 62-year-old Korean female was referred to our hospital due to chronic cough, sputum, and hemoptysis lasting more than 5 months. The patient’s sputum was positive for acid-fast bacilli staining and nontuberculous mycobacteria (NTM) were isolated twice from sputum specimens. The isolate was identified as *M. abscessus* subsp. *abscessus*. The isolate had a point mutation of C → T at position 19 (C19 → T) in the *erm*(41) gene, instead of expected C28 sequevar of *erm*(41), and had no *rrl* mutation. The isolate displayed a clarithromycin susceptible phenotype with an Arg → Stop codon change in *erm*(41). The patient was successfully treated with a macrolide-containing regimen.

**Conclusion:**

This is the first case of pulmonary disease caused by a strain of *M. abscessus* subsp. *abscessus* showing clarithromycin susceptible phenotype due to T19 sequevar of the *erm*(41) gene. The *erm*(41) gene is clinically important, and non-functional *erm* alleles may be an important issue for the management of MABC lung disease. The presence of a non-functional *erm*(41) allele in *M. abscessus* subsp. *abscessus* isolates may be associated with better outcomes.

## Background

The prevalence of lung diseases caused by nontuberculous mycobacteria (NTM) is increasing worldwide [1, 2]. *Mycobacterium abscessus* complex (MABC) is a rapidly growing mycobacterium and is the second most common cause of NTM lung disease after *M. avium* complex in many countries [3–5]. In addition, MABC has emerged as an important pathogen in patients with cystic fibrosis and chronic lung diseases, such as bronchiectasis [6–10].

MABC is the most drug resistant of mycobacterial pathogens, resulting in limited therapeutic options and a high treatment failure rate [11–14]. MABC is comprised of three closely related subspecies: *M. abscessus* subsp. *abscessus*, *M. abscessus* subsp. *massiliense* and *M. abscessus* subsp. *bolletii* [15, 16]. *M. abscessus* subsp. *abscessus* encodes a functional erythromycin ribosomal methylase gene, *erm*(41), which modifies the binding site for macrolide antibiotics, causing inducible macrolide resistance [17–19]. However, some clinical isolates of *M. abscessus* subsp. *abscessus* were susceptible to macrolide antibiotics, even after extended incubation of 14 days [20]. Loss of function of *erm*(41) genes was associated with a T-to-C substitution at position 28 of the gene (T28C), leading to an amino acid change from Trp to Arg at codon 10 [17, 18]. Pulmonary disease caused by these strains with *M. abscessus* subsp. *abscessus* with C28 sequevar may be responsive to macrolide-containing antibiotic regimens [20]. Conversely, all *M. abscessus* subsp. *abscessus* strains with a T28 sequevar were thought to be resistant to clarithromycin with extended incubation [18].

Although there were multiple polymorphisms associated with amino acid changes, only this T28C substitution resulted in loss of *erm*(41) gene function and no other nucleotide substitution was known to be associated with macrolide susceptibility [20]. We report the first case of pulmonary disease caused by a strain of *M. abscessus* subsp. *abscessus* that was susceptible to clarithromycin due to a T19 sequevar of the *erm*(41) gene. The patient was treated successfully with macrolide-containing antibiotics. This study was approved by the institutional review board of the Samsung Medical Center.

## Case presentation

**Fig. 1:**
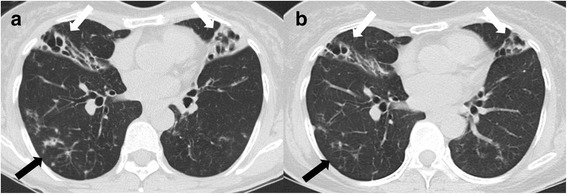
A 62-year-old female with bronchiectasis and nontuberculous mycobacterial lung disease caused by *Mycobacterium abscessus* subsp. *abscessus*. **a** Transverse chest computed tomography scan (2.5-mm-section thickness) at the start treatment revealed bilateral bronchiectasis and consolidations (white arrows) in the right middle lobe and lingular division of the left upper lobe as well as multiple tree-in-bud appearances (black arrow), suggesting bronchiolitis. **b** Transverse chest computed tomography scan (2.5-mm-section thickness) at 12 months of antibiotic treatment revealed decreased consolidation around the bronchiectasis (white arrows) and decreased bronchiolitis (black arrow)

A 62-year-old Korean female was referred to our hospital due to chronic cough, sputum, and hemoptysis lasting more than 5 months. She was a non-smoker and had no history of previous treatment for pulmonary tuberculosis. The patient was 149.7 cm tall and weighed 40.7 kg. The erythrocyte sedimentation rate was 77 mm/h, and C-reactive protein was 2.37 mg/dL. A human immunodeficiency virus antibody test was negative. A chest computed tomography (CT) scan revealed bronchiectasis and bronchiolitis in both lungs, suggesting the nodular bronchiectatic form of NTM lung disease (Fig. 1a).

The patient’s sputum was positive for acid-fast bacilli staining and NTM were isolated twice from sputum specimens in both solid (3 % Ogawa solid media; Shinyang, Seoul, South Korea) and liquid culture system (Bactec MGIT 960 system; BD Diagnostics, Sparks, MD, USA). To identify an etiological agent, bacteria grown in the MGIT 960 culture system were initially propagated in 7H9 broth (Difco Laboratories, Detroit, MI, USA) supplemented with 10 % (vol/vol) oleic acid-albumin-dextrose-catalase (OADC; BD Diagnostics) for 7 days at 37 °C. They were then sub-cultured in egg-based 3 % Ogawa solid media (Shinyang, Seoul, South Korea), and genomic DNA was extracted from cultured bacteria. *M. abscessus* was the initial species identified using a reverse line blot hybridization assay (REBA Myco-ID; M&D, Inc., Wonju, South Korea) based on the *rpoB* gene [21].

To confirm the accuracy of this identification, sequencing analyses of *rpoB*, *hsp65*, and 16S rRNA were performed using GenBank (http://blast.ncbi.nlm.nih.gov/) with the BLAST algorithm [22–24]. The 16S rRNA sequences were 100 % identical to *M. abscessus* subsp. *abscessus* (GenBank accession no. NR074427), *M. abscessus* subsp. *massiliense* (GenBank accession no. NR074421), *M. chelonae* (GenBank accession no. AY457082), and *M. abscessus* subsp. *bolletii* (GenBank accession no. NR043236). The *rpoB* sequences showed 99.7 % similarity to those of the *M. abscessus* subsp. *abscessus* type strain, with only a 2-base mismatch (GenBank accession no. CU458896). The *hsp65* sequences were 100 % identical to those of the *M. abscessus* subsp. *abscessus* type strain (GenBank accession no. CU458896). The isolate was identified as *M. abscessus* subsp. *abscessus* by sequencing based method.

Drug susceptibility testing was performed using a broth microdilution method and *M. peregrinum* ATCC 700686 was used for quality control according to the guidelines [25], revealing that the isolate was susceptible to clarithromycin (minimum inhibitory concentration [MIC], ≤0.5 μg/mL), even after extended incubation for 14 days (Table 1). The isolate was genotyped for *erm*(41) polymorphism and for *rrl* mutation, which are known as the main mechanisms of macrolide resistance [26]. The isolate had a point mutation of C → T at position 19 (C19 → T) in the *erm*(41) gene, instead of the expected C28 sequevar of *erm*(41). It also had no *rrl* mutation. To the best of our knowledge, the C19 → T mutation of *erm*(41) in *M. abscessus* subsp. *abscessus* is the first description.

**Table 1:** Drug susceptibility testing results for antimicrobial agents against the isolate

Drug	MIC (μg/mL) for each category			MIC of isolate (μg/mL)
	Susceptible	Intermediate	Resistant	
Amikacin	≤16	32	≥64	8
Cefoxitin	≤16	32–64	≥128	64
Imipenem	≤4	8–16	≥32	8
Clarithromycin	≤2	4	≥8	≤0.5
Ciprofloxacin	≤1	2	≥4	>16
Moxifloxacin	≤1	2	≥4	16
Doxycycline	≤1	2–4	≥8	>32

The patient received oral clarithromycin (1,000 mg/d) with an initial 4-week hospitalization for intravenous amikacin and cefoxitin [27, 28]. At day 20 of treatment, clarithromycin was switched to azithromycin (250 mg/d) due to gastrointestinal disturbance. After discharge, the patient received oral azithromycin for a total duration of 15 months. Her sputum cultures converted to and remained negative after 2 months of antibiotic treatment. Chest CT at 12 months of treatment revealed improvement in consolidations and bronchiolitis (Fig. 1b).

## Discussion

It is important to distinguish the three subspecies of MABC because of their differences in susceptibility to clarithromycin [29–32]. *M. abscessus* subsp. *abscessus*, *M. abscessus* subsp. *massiliense*, and *M. abscessus* subsp. *bolletii* are commonly inducible resistant, susceptible, and resistant to clarithromycin, respectively [15].

**Fig. 2:**
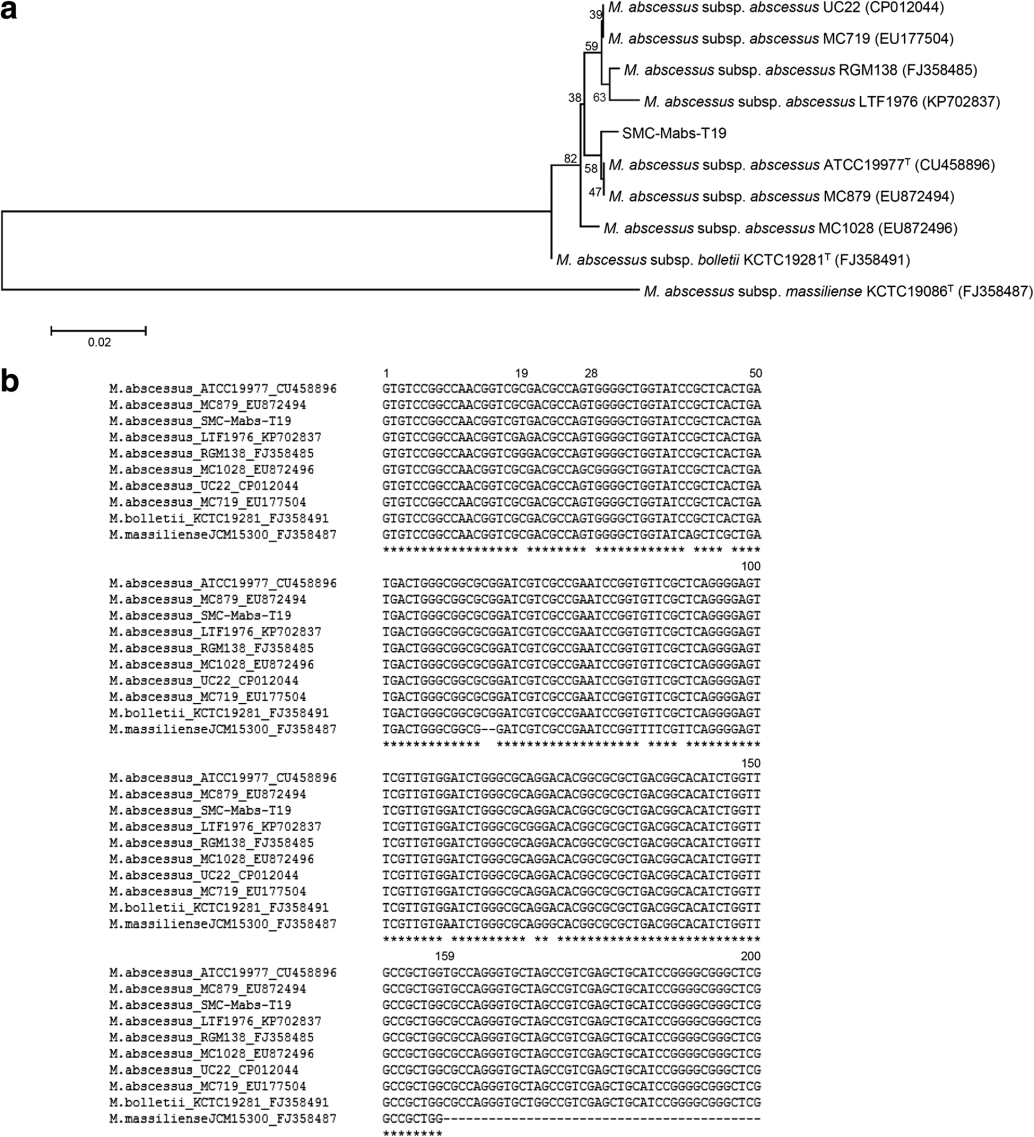
Analysis of DNA sequences in the *erm*(41) of *Mycobacterium abscessus* subsp. *abscessus* clinical isolate SMC-Mabs-T19. **a** Phylogenetic position of isolate SMC-Mabs-T19 and other strains belonging to *M. abscessus* complex based on entire *erm*(41) gene sequences. This tree was constructed using the neighbor-joining method. Percentages indicated at nodes represent the bootstrap levels supported by 1,000 re-sampled datasets. Scale bars indicate evolutionary distance in base substitutions per site. **b** Sequence alignment of *erm*(41) from SMC-Mabs-T19 and other strains belonging to the *M. abscessus* complex. Base numbering is from the first base of *erm*(41). Identical nucleotides are indicated by an asterisk below sequences. Two deletion sites of *M. abscessus* subsp. *massiliense* are indicated by dashes

This is the first case of pulmonary disease caused by a strain of *M. abscessus* subsp. *abscessus* showing a clarithromycin susceptible phenotype due to the T19 sequevar of *erm*(41) gene. According to a previously reported paper by Nash et al., *M. abscessus* subsp. *abscessus* strains with T28 → C had no inducible resistance to clarithromycin and had low MIC. In the present study, the *M. abscessus* subsp. *abscessus* clinical isolate had a C-to-T mutation at position 19 (C19 → T) leading to an Arg → Stop codon change at codon 7 of the *erm*(41) gene. This strain was named SMC-Mabs-T19. The SMC-Mabs-T19 strain revealed low MIC, similar to the *M. abscessus* subsp. *abscessus* strain with a C28 sequevar. Therefore, maintenance of low clarithromycin MIC against the SMC-Mabs-T19 strain might have been a result of the production of non-functional Erm(41). The entire *erm*(41) sequence of the susceptible isolate SMC-Mabs-C19 was unique and differed from that of *M. abscessus* subsp. *abscessus* type strain (GenBank accession no. CU458896) by only two bases: a C-to-T mutation at position 19 (C19 → T), and a T-to-C mutation at position 159 (T159 → C) (Fig. 2a and b). Of these differences, T159 → C was also present in the inducible resistant strains MC719 and UC22 (GenBank accession nos. EU177504 and CP012044, respectively). Therefore, the T19 sequevar was the most likely explanation for the lack of function of *erm*(41) alleles from the SMC-Mabs-T19 strain. Until now, *erm*(41) with a T19 sequevar has not been reported in GenBank. However, we found only two *M. abscessus* subsp. *abscessus* clinical isolates harboring *erm*(41) with C19 → G or A point mutations, and more information regarding drug susceptibility was not available (GenBank accession nos. FJ358485 and KP702837, respectively; Fig. 2b).

## Conclusions

The *erm*(41) gene is clinically important, and non-functional *erm* alleles may be an important issue for management of MABC lung disease. The presence of a non-functional *erm*(41) allele in *M. abscessus* subsp. *abscessus* isolates may be associated with better outcomes.

### Ethics and consent to participate

This study protocol was approved by the institutional review board of the Samsung Medical Center (IRB approval 2008-09-016).

### Consent to publish

Written informed consent was obtained from the patient for publication of this Case report and any accompanying images. A copy of the written consent is available for review by the Editor of this journal.

### Availability of data and materials

All the data supporting the findings is contained within the manuscript.

## Abbreviations

CT: computed tomography
*erm*(41): erythromycin ribosomal methylase gene
MABC: *Mycobacterium abscessus* complex
NTM: nontuberculous mycobacteria
